# Congenital Spigelian Hernia With Ipsilateral Ectopic Testis

**DOI:** 10.31486/toj.21.0134

**Published:** 2022

**Authors:** Akhilesh Gonuguntla, Sundeep Payyanur Thotan, Nitin Pai, Vijay Kumar, Santosh Padubidri Prabhu

**Affiliations:** ^1^ Kasturba Medical College, Manipal Academy of Higher Education, Manipal, Karnataka, India; ^2^ Department of Pediatric Surgery, Kasturba Medical College, Manipal Academy of Higher Education, Manipal, Karnataka, India

**Keywords:** *Cryptorchidism*, *herniorrhaphy*, *infant*, *laparoscopy*, *orchiopexy*, *spigelian hernia*

## Abstract

**Background:** The association between congenital spigelian hernia and ipsilateral ectopic testis has been debated in the literature, and the management of such cases has yet to be standardized. Both pediatric surgeons and sonographers should be aware of this entity to allow for prompt diagnosis and monitoring/repair because congenital spigelian hernias have a high risk of incarceration.

**Case Report:** A 3-month-old male presented with left-sided abdominal wall swelling present since birth with coexisting left-sided undescended testis. Ultrasound confirmed the suspicion of a congenital spigelian hernia with undescended testis but failed to locate the ectopic testis. Diagnostic laparoscopy and open repair of the spigelian hernia were performed when the patient was 9 months of age. Left-sided orchidopexy was also performed as the left testis was located within the spigelian sac. The patient was asymptomatic at 1-year follow-up.

**Conclusion:** The association between congenital spigelian hernia and ipsilateral ectopic testis requires the surgeon and sonographer to pay special attention to the spigelian hernia sac as it may contain the ectopic testis. Orchidopexy and hernia repair in very young children may be delayed while closely monitoring for incarceration to allow for improvement in immunity, an increase in size of the spermatic cord and vasculature, and avoidance of the stress of 2 separate surgeries. The surgical approach can be laparoscopic or open depending on the experience of the surgeon and the complexity of intraoperative findings.

## INTRODUCTION

With fewer than 100 reported cases in the English literature from 1900 to 2015, spigelian hernias are a rare variety of abdominal wall hernia in the pediatric population.^1^ Congenital spigelian hernias in males are a topic of discussion because of their association with ipsilateral undescended testis/ectopic testis in 40% to 80% of cases.^1-5^ Increasing reports of this association have led to dialog among pediatric surgeons on whether the relationship is syndromic, sequential, or merely coincidental.^1,5^ A high degree of suspicion is required when an anterior abdominal wall hernia presents with an impalpable, ipsilateral undescended testis because without intervention, the risk of incarceration is high (17%-24%) in cases of spigelian hernia.^6,7^ Misdiagnosis in uncomplicated cases can result in a delay in management and a potential increase in morbidity.^2,8^ We report our experience managing a congenital spigelian hernia with ipsilateral ectopic testis in a 3-month-old male and discuss the origin of the association with relevant clinical pearls.

## CASE REPORT

A 3-month-old male presented with an abdominal wall swelling in the left flank that was reported to have gradually enlarged since birth. The swelling was associated with a transient increase in size during episodes of crying or coughing followed by spontaneous reduction. The infant was born at term by elective caesarean section to a gravida 2 para 1 (1 live birth) mother with gestational diabetes mellitus.

Examination revealed a soft, reducible swelling with an expansile impulse when the infant cried that was located in the posterolateral aspect of the left lower abdominal wall and extended anteriorly onto the left iliac fossa. The left hemiscrotum was empty, and the left testis was not palpable. A provisional diagnosis of a congenital spigelian or lumbar hernia with left undescended testis was considered.

Ultrasound of the abdomen revealed 2 abdominal wall defects. The first fascial defect was in the anterior abdominal wall in the left iliac fossa and measured 1.6 cm with a sac extending laterally and measuring 2.8 × 1.2 × 3 cm. A second fascial defect was noted posterosuperior to the first defect in the posterior abdominal wall lateral to the kidney that measured 1.3 × 0.6 cm with a sac measuring 2.7 × 1.2 × 3 cm. The left testis was not visualized on ultrasound, while the opposite side had no abnormalities, consistent with the clinical examination and the initial diagnosis. Diagnostic laparoscopy and open repair were planned for when the infant was 9 months of age.

**Figure 1.**
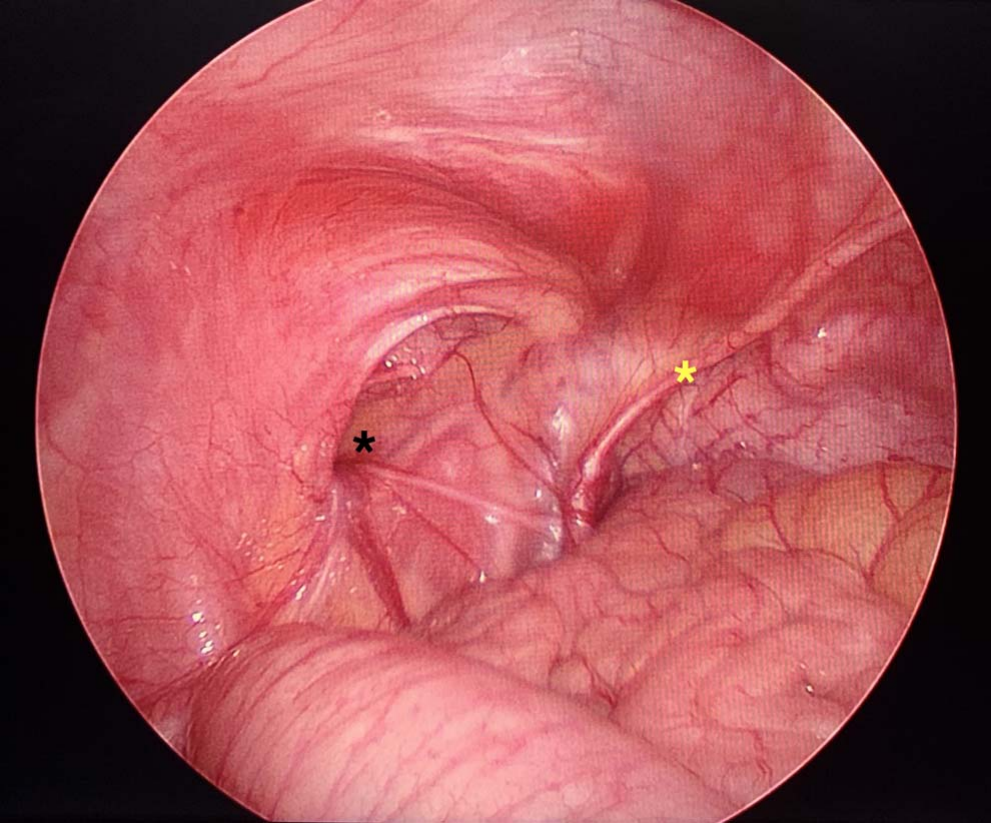
Laparoscopic view of the defect. The asterisk on the left represents the vas and vessels at the lateral aspect of the spigelian defect. The asterisk on the right represents the lateral umbilical fold.

When the patient reached the age of 9 months, diagnostic laparoscopy revealed 2 apparent fascial defects. The larger defect, with clearly defined margins and measuring approximately 2.5 × 2 cm, was present in the lower left abdominal wall approximately 1.5 cm lateral to the lateral umbilical ligament. The sac proceeded anteriorly and then superolaterally in the subcutaneous plane in the anterolateral abdominal wall. The left undescended testis was identified in the hernia sac with the vas deferens and the accompanying vasculature entering the sac at the lateral end of the fascial defect (Figure 1). The gubernaculum and inguinal canal were absent on the left side. This hernial defect appeared to be consistent with a spigelian defect.

The second potential fascial defect was noticed posterosuperiorly as a depression in the parietal muscle bed. However, neither clear margins nor a hernia sac was seen with this fascial defect as identified in the ultrasound. These findings led to the confirmation of the diagnosis of congenital spigelian hernia with ectopic testis.

**Figure 2.**
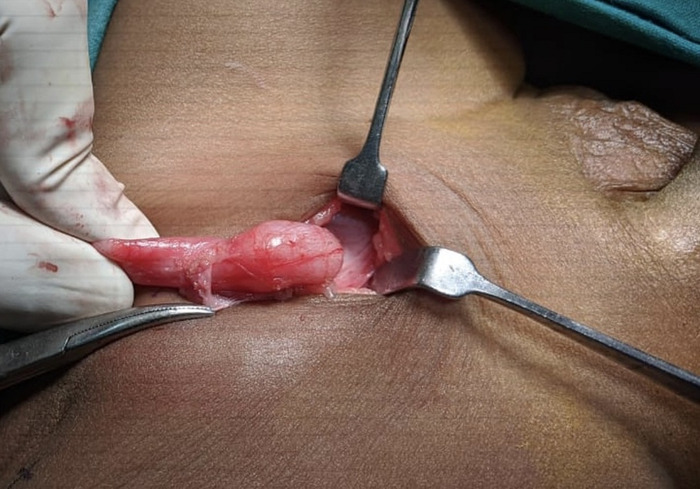
Ectopic testis with sac in the subcutaneous plane.

The hernia sac from the first fascial defect was isolated and separated from the testis, vas, and vessels. Although 80% of the size of the opposite testis, the consistency and contour of the left testis were normal. In addition, the length of the vasculature was adequate. A herniotomy was performed flush with the abdominal wall. The testis was rerouted medial to the inferior epigastric vessels and tunneled through the subcutaneous tissue into the scrotum. Left-sided orchidopexy was performed in the conventional manner (Figure 2). The muscular defect was then closed with interrupted polypropylene sutures. Mesh was avoided as the muscular edges could be approximated without any tension. The second potential fascial defect was not repaired because only a depression was identified instead of a clearly defined defect with margins. Additionally, no sac was seen, and the saucer-like depression was wide, precluding the occurrence of complications. The sac seen on ultrasound associated with the posterolateral fascial defect was presumed to be the sac of the first hernial defect. The child recovered uneventfully and was asymptomatic at 15-day and 1-year follow-ups.

## DISCUSSION

Spigelian fascia is the aponeurotic part of the transversus abdominis muscle between the lateral border of the rectus abdominis muscle and the semilunar line extending from the ninth costal cartilage to the pubic tubercle. The semilunar line marks the transition between muscle and aponeurosis in the internal oblique muscle.^7^ Most commonly, the spigelian hernia sac protrudes through defects in the transversus abdominis aponeurosis and internal oblique muscle to lie beneath the overlying thick, intact external oblique aponeurosis below the level of the arcuate line.^7^ Fibers of the transversus abdominis aponeurosis and internal oblique muscle run parallel to each other below the umbilicus, allowing for the development of potential weak points through which a hernia could develop.^7,9^ In addition, spigelian hernias are commonly visible lateral to the confluence of the arcuate and semilunar lines because the hernial sac grows laterally as it is limited medially by the rectus sheath and muscle.^7,10^ Spigelian hernias are classified based on their relation to the inferior epigastric vessels into the classic (superolateral to vessels) and low types (inferomedial to vessels).^2,7^ According to Sengar et al, the low variety is more commonly associated with congenital ipsilateral cryptorchidism and is commonly misdiagnosed as direct inguinal hernia.^2^ Early differentiation of spigelian hernias from other hernias is essential, as the risk of incarceration is notably higher with spigelian hernias, and misdiagnosis could delay management.^2^ However, our patient presented with the classic type in conjunction with an undescended testis.

Unlike adult spigelian hernias, which are usually attributable to a chronic increase in intra-abdominal pressure or trauma and have a female preponderance, up to 80% of spigelian hernias in children are thought to be nontraumatic in origin with a male preponderance (3-4:1).^1,2^ While up to one-third of all spigelian hernias are associated with other anomalies including myelomeningocele, anorectal malformations, and skeletal abnormalities, up to 80% of spigelian hernias in males are associated with an ectopic testis.^1,11^ Up to 87% of the time, the testis is present in the hernia sac.^11,12^ Although physical examination is the mainstay of diagnosis, ultrasound is a useful adjunct to evaluate the hernia prior to surgery and to locate the testis.^3,8,11-13^ However, in our case, the left testis was not palpable by clinical examination and not visualized by ultrasound. Other authors have reported a similar failure of ultrasound to locate the testis on the side of the spigelian hernia.^4,14,15^ Consequently, the sonographer must maintain a high index of suspicion when suspecting a spigelian hernia in a patient with undescended testis and attempt to locate the testis in the hernia sac.^14^ Even if ultrasound fails to demonstrate the testis, the surgeon should be prepared to locate it in the hernia sac intraoperatively.^12^ Because of the nature of the spigelian sac to grow in the superolateral direction and the propensity of the neck to lie near the inguinal ring, the sonographer must also keep in mind that a spigelian hernia could be mistaken for other hernias such as an inguinal hernia or a lumbar hernia.

To delineate the anatomy of the surgical site, we recommend a diagnostic laparoscopy before performing the orchidopexy and open hernia repair.^13^ Special attention must be paid to the location and size of the defect, length of the testicular vasculature, and presence of the inguinal canal and gubernaculum. The location of the defect helps classify the hernia as a classic or a low hernia. The size of the defect determines whether herniorrhaphy or hernioplasty is required. Durham and Ricketts placed an SIS mesh (Cook Biotech), while Singal et al utilized a VYPRO (Vicryl and Prolene) mesh to repair the hernia as the defect was too large for a herniorrhaphy.^3,4^ Both authors reported good results with hernioplasty and neither reported recurrence in their cases. If the length of the testicular vasculature is sufficient, a 1-stage orchidopexy can be performed. In most cases, including our own, the length of the testicular vessels was sufficient. No cases of spigelian hernia-ectopic testis have been reported in which an ipsilateral gubernaculum or inguinal canal was confirmed by the surgeon, but in at least 69% of spigelian hernia-undescended testis cases, the ipsilateral gubernaculum and/or inguinal canal was absent.^1^ The absence of the gubernaculum and inguinal canal, the adequate length of the testicular vessels, and the location of the defect of the spigelian hernia play an important role in hypothesizing the pathophysiology of this embryologic association.

Since the late 1990s, the association between congenital spigelian hernias and ipsilateral undescended testis has been increasingly highlighted by many authors,^1-5,8-16^ leading to discussions to establish the most suitable hypothesis for the characterization of this relationship. Initially, the occurrence of spigelian hernia and ipsilateral undescended testis together was thought to be a coincidence, but as multiple case reports were published, enough evidence accrued to challenge this notion. Although no widely approved consensus has been reached among the pediatric surgery research community, multiple hypotheses have been suggested to explain where the embryologic defect occurred and whether the association between spigelian hernia and ectopic testis is a sequence or a syndrome.

Early researchers suggested that abdominal wall defects may impair the generation of sufficient intra-abdominal pressure that mechanically drives the descent of the testis along with the gubernaculum.^1^ Raveenthiran criticized this theory, as the defect in the abdominal wall is very small in most cases and unlikely to result in a significant decrease in intra-abdominal pressure.^16^ Silberstein et al and Al-Salem reported the absence of the inguinal canal in their cases and hypothesized that the testis takes the path of least resistance through the congenital defects in the spigelian fascia.^9,10^ This theory was challenged by several, including Raveenthiran, who believed that for unknown reasons, maldescent of the testis would occur, dragging a processus vaginalis during descent and opening a defect in the aponeurotic layers.^14-16^ Raveenthiran reiterated that this potential point of herniation would be opened by increased intra-abdominal pressure caused by other comorbidities.^15,16^ However, Rushfeldt et al argued that this explanation did not reflect general embryologic knowledge and that the testis could not drag down a processus vaginalis.^12^ Rushfeldt et al proposed that the failure of the gubernaculum and, hence, the inguinal canal to form would arrest the descent of the testis, leading it to induce the formation of a spigelian hernia as an “emergency exit.”^12^ Jones and Hutson disagreed with the hypothesis of Rushfeldt et al because if an undescended testis could induce rescue canals, the incidence of spigelian hernia should approach the incidence of undescended testis, which is untrue.^1^ Rushfeldt et al hypothesized that the “Spigelian-cryptorchidism syndrome” was characterized by a congenital ipsilateral defect in the spigelian fascia, hernia sac with testis, absence of gubernaculum, and absence of inguinal canal, a hypothesis that several authors accepted.^5,12,14^ Jones and Hutson agreed with Raveenthiran and attempted to explain the unknown reason behind his theory via the analysis of various published animal models on testicular descent.^1^ They suggested that the gubernaculum becomes located slightly superior to the usual site of attachment toward the scrotal sac, which corresponds to the site of the future spigelian hernia. This orientation results in the migration of the testis into the anterior abdominal wall through the spigelian fascia, leading to the manifestation of an ectopic testis. However, as the genitofemoral nerve plays an essential role in maintaining the gubernaculum but is located further caudally, the gubernaculum loses the trophic support of the nerve and degenerates.^1^ Hence, this theory explains the absence of the gubernaculum and the inguinal canal, the presence of ectopic testis in the spigelian hernia sac, and the adequate length of testicular vasculature. Although the theory is relatively new with support from only animal models, it received the support of Sengar et al, who believe this theory explains their findings of a higher incidence of low spigelian hernias in association with undescended testis.^2^ We agree with Raveenthiran^15,16^ and Jones and Hutson,^1^ as inherent defects in both the testis and gubernacular orientation appear to explain all the features of the syndrome proposed by Rushfeldt et al.^12^ Hormonal crosstalk between the androgen-producing cells of the testis, gubernaculum, and the genitofemoral nerve appears to be critical in testicular descent.^1^ In particular, testosterone appears to influence the genitofemoral nerve to secrete calcitonin gene-related peptide that mediates gubernacular migration during testicular descent.^1^ The malfunction of this testosterone-mediated process would explain the features of the spigelian hernia-ectopic testis syndrome. However, the presentation of a classic spigelian hernia with ectopic testis in the sac deviates from the Sengar et al hypothesis and suggests that the Jones and Hutson hypothesis may explain both low and classic spigelian hernia-ectopic testis.^1,2^

Some authors question the ideal time to operate.^11,13,14^ Vascular damage, tension on and compression of the spermatic cord structures, and scrotal infection are possible complications of surgery and may result in testicular atrophy. Some authors propose that spigelian hernia repair surgery should be done immediately to limit incarceration and that orchidopexy should be delayed until the patient is 1 year old to allow for immunity to improve and for the vas deferens and vasculature to enlarge and attain more length and girth.^11,14^ Inan et al suggested the conservative approach of waiting until the patient is 1 year old while monitoring for incarceration.^14^ We operated on our patient at 9 months of age while closely monitoring for incarceration in the interim between diagnosis and repair. We recommend the approach of intervention at an older age while monitoring for incarceration to avoid the increased stress and risk of potential surgery-related complications associated with 2 separate procedures compared to a single surgery.

In uncomplicated cases, complete laparoscopic repair may be a viable option.^17^ Deshmukh et al reported successful results with laparoscopic orchidopexy and repair for spigelian hernia-ectopic testis.^17^ However, more data are required to definitively recommend complete laparoscopic repair vs the conventional open approach. Hence, as a prudent approach, we recommend that this type of case be approached laparoscopically and converted to an open approach if required, depending on the complexity of operative findings and the surgeon's experience.

## CONCLUSION

Regardless of the nature of the relationship between spigelian hernia and ipsilateral ectopic testis, the association is an important consideration for pediatric care providers and sonologists to prevent delayed diagnosis and management. In particular, health care providers should expect to find the ectopic testis in the spigelian hernia sac even if the testis is not clinically or radiologically located. When the condition is diagnosed in early infancy, we recommend that surgery be delayed while monitoring for incarceration to allow for improvement in immunity, an increase in size of the spermatic cord and vasculature, and avoidance of the stress of 2 separate surgeries. Although no study has compared open and laparoscopic approaches for repairing spigelian hernia with ectopic testis, we recommend that this decision be made based on the complexity of intraoperative findings and the surgeon's level of experience.

## Notes

*This article meets the Accreditation Council for Graduate Medical Education and the American Board of Medical Specialties Maintenance of Certification competencies for Patient Care and Medical Knowledge.*
